# SARS-CoV-2 Cross-Reactivity in Prepandemic Serum from Rural Malaria-Infected Persons, Cambodia

**DOI:** 10.3201/eid2802.211725

**Published:** 2022-02

**Authors:** Jessica Manning, Irfan Zaidi, Chanthap Lon, Luz Angela Rosas, Jae-Keun Park, Aiyana Ponce, Jennifer Bohl, Sophana Chea, Maria Karkanitsa, Sokunthea Sreng, Huy Rekol, Char Meng Chour, Dominic Esposito, Jeffery K. Taubenberger, Matthew J. Memoli, Kaitlyn Sadtler, Patrick E. Duffy, Fabiano Oliveira

**Affiliations:** National Institute of Allergy and Infectious Diseases, Bethesda, Maryland, USA (J. Manning, I. Zaidi, C. Lon, L.A. Rosas, J.-K. Park, A. Ponce, J. Bohl, J.K. Taubenberger, M.J. Memoli, P.E. Duffy, F. Oliveira);; National Center for Parasitology, Entomology and Malaria Control, Phnom Penh, Cambodia (S. Chea, S. Sreng, H. Rekol, C.M. Chour);; National Institute of Biomedical Imaging and Bioengineering, Bethesda (M. Karkanitsa, K. Sadtler);; Frederick National Laboratory for Cancer Research, Frederick, Maryland, USA (D. Esposito)

**Keywords:** malaria, SARS-CoV-2, serosurvey, Cambodia, cross-reactivity, severe acute respiratory syndrome coronavirus 2, viruses, vector-borne infections, zoonoses, COVID-19, coronavirus disease

## Abstract

Inhabitants of the Greater Mekong Subregion in Cambodia are exposed to pathogens that might influence serologic cross-reactivity with severe acute respiratory syndrome coronavirus 2. A prepandemic serosurvey of 528 malaria-infected persons demonstrated higher-than-expected positivity of nonneutralizing IgG to spike and receptor-binding domain antigens. These findings could affect interpretation of large-scale serosurveys.

Serosurveys for severe acute respiratory syndrome coronavirus 2 (SARS-CoV-2) in the Greater Mekong Subregion (GMS) of Cambodia have been limited to those screening healthcare workers in 2 urban hospital-based settings (*1*,*2*). These antibody-based studies are necessary to determine at-risk populations and direct disease containment measures; however, before informing public health decisions, serologic assays require careful, country-specific calibration because several regions report fluctuating results or high background reactivity in different populations (*3*–*5*). This variability might be attributable to myriad serologic assays, the hypothesized cross-reactivity from common cold–type respiratory coronaviruses (*6*), previous *Plasmodium* infections (*7*,*8*; S. Lapidus et al., unpub. data, https://www.medrxiv.org/content/10.1101/2021.05.10.21256855v1), or previously uncharacterized betacoronaviruses in wildlife populations in the rural GMS (*9*–*11*). Although many serologic SARS-CoV-2 investigations are in progress, considering how pathogen diversity in the GMS might influence estimations of SARS-CoV-2 seroprevalence is prudent.

## The Study

We tested serum or plasma samples collected from 528 malaria-infected persons in Cambodia during 2005–2011 (before SARS-CoV-2 emerged in 2019) for IgG reactive to SARS-CoV-2 spike and receptor-binding domain (RBD) proteins by using ELISA (*12*,*13*). We used de-identified, anonymized serum or plasma samples biobanked after malaria research studies (NCT00341003, NCT00663546, and NCT01350856, approved by the National Institute of Allergy and Infectious Diseases and the National Ethics Committee on Human Research in Cambodia) for this retrospective study.

**Figure 1:**
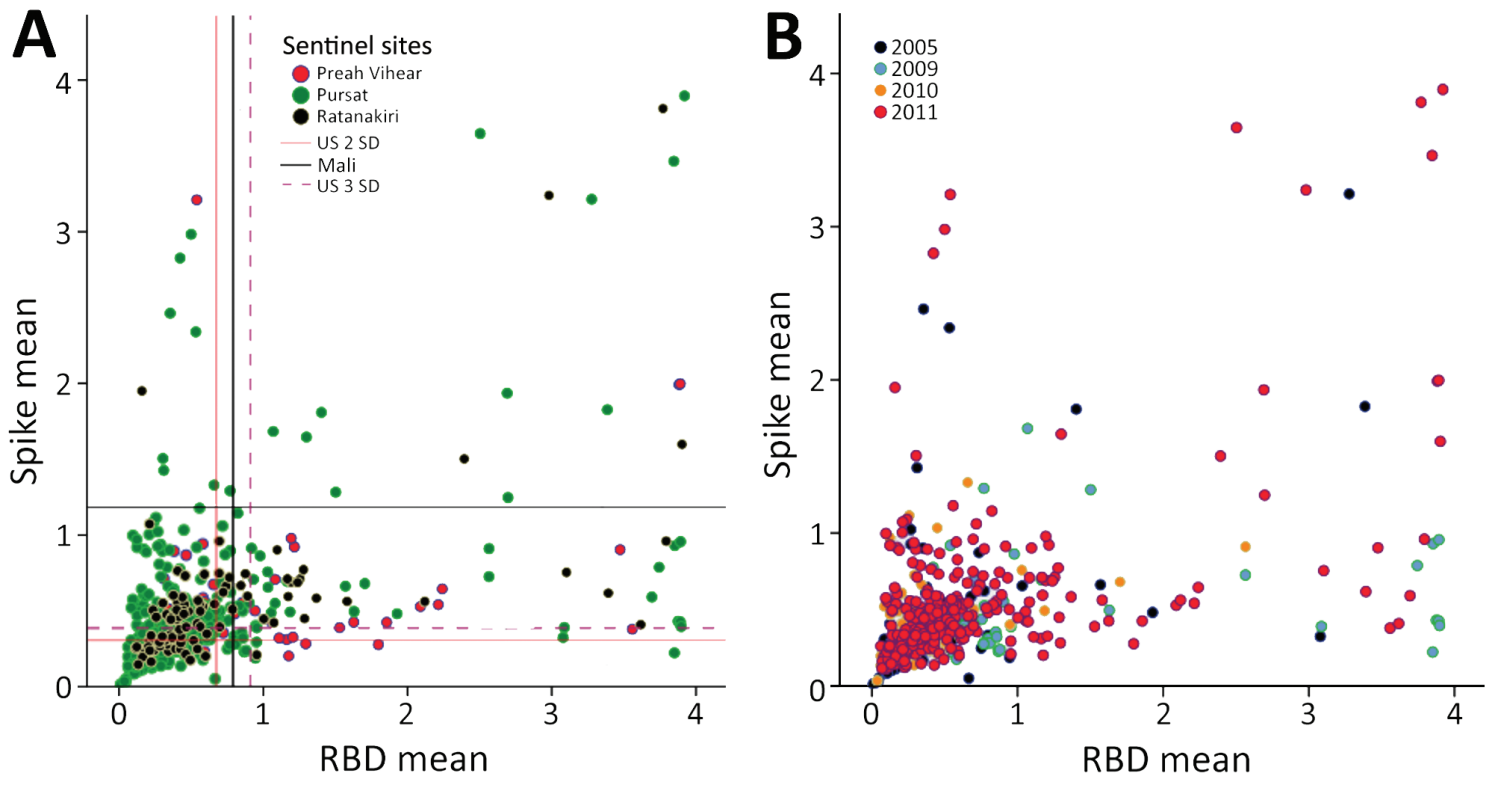
Mean antibody intensity in arbitrary ELISA units to spike and RBD in serum samples from prepandemic, malaria-positive rural persons in Cambodia, 2005–2011. A) Provinces indicated by color: Preah Vihear (pink), Pursat (green), Ratanakiri (black). B) Years indicated by color: 2005 (purple), 2009 (turquoise), 2010 (orange), and 2011 (pink). RBD, receptor binding domain.

Because 6 other coronaviruses (OC43, HKU1, 229E, NL63, severe acute respiratory syndrome coronavirus 1 [SARS-CoV-1], and Middle East respiratory syndrome coronavirus) possess structural proteins capable of infecting humans, we selected highly specific ELISAs for the SARS-CoV-2 structural proteins (*12*,*13*). Compared with other coronaviruses, SARS-CoV-2 shows varying levels of spike protein sequence homology; levels are highest for SARS-CoV-1 (76% identity, 87% similarity) and lowest for the common cold coronavirus HKU1 (29% identity, 40% similarity) (*12*). Reactivity to both spike and RBD antigens above cutoff values is required for a positive test with reported sensitivity of 100% (95% CI 92.9%–100%) and specificity of 100% (95% CI 98.8%–100%) (*12*,*13*). Prepandemic samples had levels above the set cutoffs for SARS-CoV-2 spike and RBD antigens (Figure 1) varying from 4.4% to 13.8% positivity to both SARS-CoV-2 spike and RBD depending on which cutoff values (calibrated for the Mali or US populations) were used for this assay (*4*,*12*,*13*) (Table; Figure 1; Appendix Table 1).

**Table:** SARS-CoV-2 ELISA results by cutoff values in prepandemic serum samples from rural malaria-experienced persons in 3 Cambodia provinces, 2005–2011*

Province	Year	Total	No. positive by 2 SDs	No. positive by 3 SDs	No. positive, Mali
Preah Vihear	2011	81	12 (15)	6 (7)	5 (6)
Pursat	2005	80	8 (10)	4 (5)	3 (4)
	2009	76	12 (16)	6 (8)	3 (0.9)
	2010	81	5 (6)	3 (4)	1 (0.3)
	2011	110	17 (15.5)	12 (11)	6 (5.4)
	Subtotal	347	42 (12)	25 (7)	13 (3.7)
Ratanakiri	2011	100	19 (19)	6 (6)	5 (5)
Total	All	528	73 (13.8)	37 (7)	23 (4.4)

*Values are no. (%) except as indicated. Using United States arbitrary ELISA unit cutoffs of 2 SDs for spike (0.674) and receptor binding domain (RBD) (0.306); United States 3 SDs for spike (0.910) and RBD (0.387); and Mali cutoff for spike (0.791) and RBD (1.183). SARS-CoV-2, severe acute respiratory syndrome coronavirus 2.

**Figure 2:**
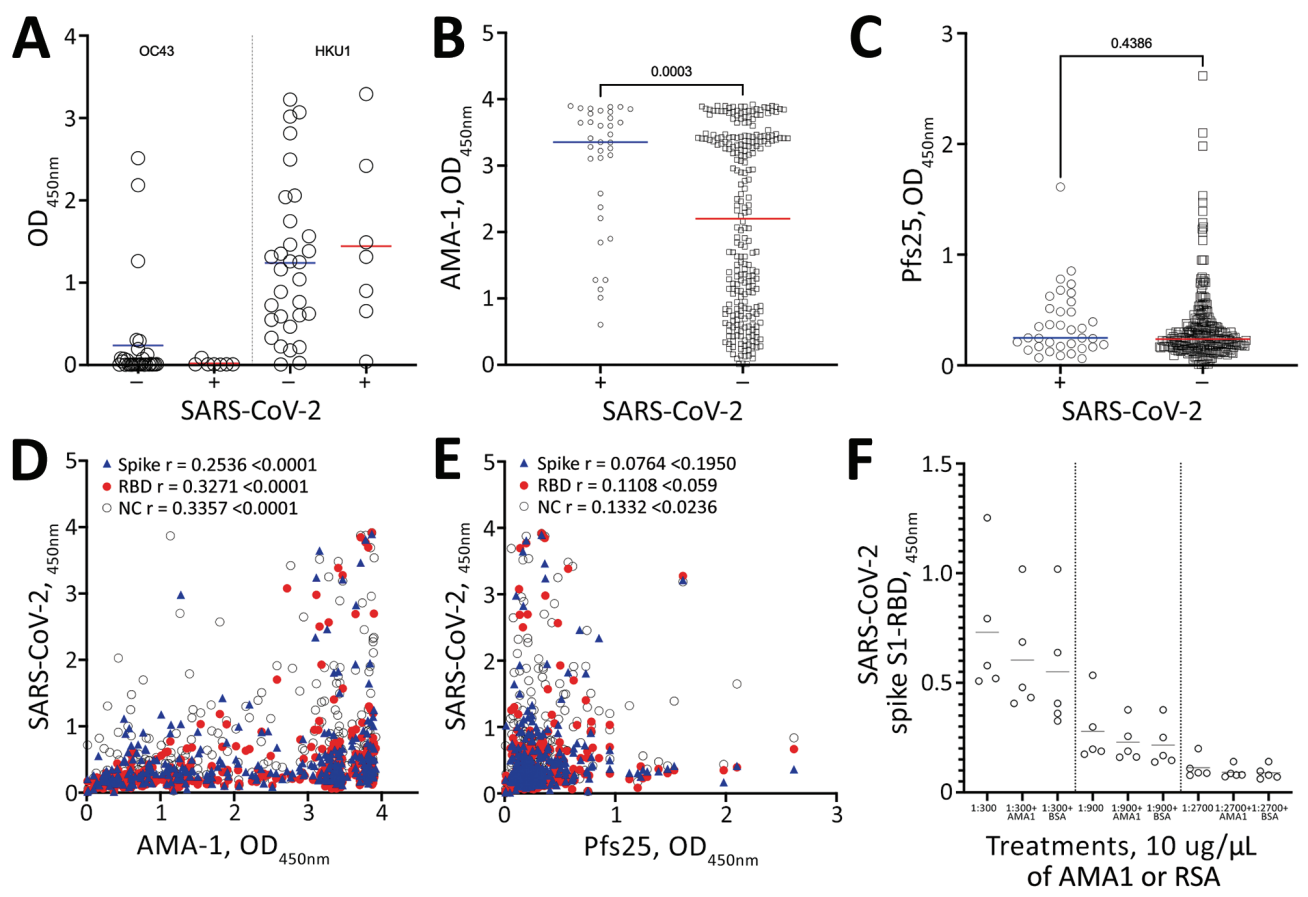
Mean antibody levels in prepandemic serum samples from malaria-positive rural persons in Cambodia, 2005–2011, to A) common cold OC43 and HKU1 viruses, B) *Plasmodium falciparum* AMA-1 and C) *P. falciparum* Pfs25 protein by SARS-CoV-2 serosurvey statuses. D–E) Correlation of mean IgG levels of AMA-1 and Pfs25 against Spike (blue triangles), RBD (red circles) and NC (open circles) IgG levels in prepandemic serum samples from malaria-positive rural persons in Cambodia. F) OD levels of RBD protein after preincubation of serum samples with 10mg/mL of AMA-1 or BSA. AMA-1, apical membrane antigen 1; BSA, bovine serum albumin; NC, nucleocapsid; OD, optical density; RBD, receptor binding domain; SARS-CoV-2, severe acute respiratory syndrome coronavirus 2.

To test whether the higher-than-expected positivity was an artifact of our in-house ELISA, we tested a subset of samples with a commercially validated SARS-CoV-2 Spike S1-RBD IgG ELISA Detection Kit (Genscript, https://www.genscript.com). Of the 24 persons who were seronegative by in-house assay and 11 who were seropositive by in-house assay, 18 tested negative and 9 tested positive by the commercial test, yielding an overall concordance of 77.1% between assays (Appendix Table 2). This inconsistency might be explained by the stringency of the in-house assay that tests both spike and RBD versus the commercial kit that tests for RBD only; nevertheless, higher-than-expected positivity was observed in both assays. Since common cold coronaviruses do circulate in Cambodia, but no cases of SARS-CoV-1 or Middle East respiratory syndrome have been documented, we tested a subset of the cohort for IgG to HKU1 and OC43. Reactivity between subjects was comparable despite SARS-CoV-2 serostatus (Figure 2, panel A).

We further tested 289 samples to assess whether a relationship existed between antibodies to *Plasmodium* spp. and SARS-CoV-2 proteins by using 2 known malarial antigens: *Plasmodium falciparum* apical membrane antigen 1 (AMA-1), which is highly immunogenic and an indicator of parasite exposure, and *P. falciparum* Pfs25 protein (Pfs25), which is poorly immunogenic and expressed only during the mosquito stages of parasite development (*4*) (Figure 2, panels B–E). Of note, when we grouped samples by SARS-CoV-2 serostatus, we detected significantly higher levels of AMA-1 antibodies in SARS-CoV-2–seropositive persons than seronegative persons (mean AMA-1 antibody level 3.0 vs. 2.1; p = 0.0003) (Figure 2, panel B). As expected, no difference was seen in antibody levels to Pfs25 with regard to SARS-CoV-2 seropositivity (Figure 2, panel C). A weak but statistically significant positive correlation was detected between spike and RBD with AMA-1 IgG (Figure 2, panel D). This finding corroborates recent observations that higher SARS-CoV-2 seroreactivity by ELISA or rapid tests is detected in persons from malaria-endemic areas, expanding previous observations to include Southeast Asia (*7*,*8*; S. Lapidus et al., unpub. data). We also evaluated samples for seroreactivity against the nucleocapsid protein that also positively correlated with the AMA-1 IgG. Only nucleocapsid antibodies were weakly correlated with Pfs25 antibodies, which reinforces the argument for nonspecific nucleocapsid reactivity (Figure 2, panel E). Preincubation with 10 mg/mL of AMA-1 or bovine serum albumin had no notable effect on reactivity to SARS-CoV-2 spike S1-RBD (Figure 2, panel F). Therefore, *Plasmodium* spp. exposure might contribute to SARS-CoV-2 malaria-related background reactivity. This reactivity could be attributed to immune responses to other *Plasmodium* spp. proteins, polyclonal B cell activation during infection, or interaction with the sialic acid moiety on N-linked glycans of the SARS-CoV-2 spike protein (*7*; S. Lapidus et al., unpub. data). Of note, SARS-CoV-2 spike proteins used in the assays were produced in HEK293 mammalian cells and likely have comparable glycosylation patterns. Elsewhere, malaria-induced cross-reactivity in prepandemic samples from malaria-experienced persons from Africa was mitigated by the modification of 2 commercial assays to add a urea wash (S. Lapidus et al., unpub. data).

To elucidate the functionality of the detected antibodies, we took a subset (n = 21) of the samples with the highest reactivity to SARS-CoV-2 total IgG and performed neutralization assays (Appendix Figure). No neutralizing activity was identified despite high levels of antibodies reacting to both spike and RBD proteins. Identical results were obtained by using a surrogate virus neutralization test targeting RBD interaction with the host cell receptor ACE2 (Genscript) (Appendix Table 3) (*14*). Both SARS-CoV-2 infection and vaccination can trigger high levels of nonneutralizing antibodies, whereas neutralizing antibodies aimed primarily at the RBD seem to wane faster and remain at low titers (*14*). Plausibly, the cross-reactive nonfunctional antibodies to SARS-CoV-2 were raised during an infection by *Plasmodium* spp. (S. Lapidus et al., unpub. data), but we cannot discard the hypothesis that nonneutralizing SARS-CoV-2–reactive antibodies in prepandemic serum samples might be linked to the ability of betacoronaviruses to evade immune recognition because of their complex surfaces (*14*,*15*). A limitation in understanding the assays’ specificity is the lack of prepandemic samples from non–malaria-endemic areas and from present-day confirmed SARS-CoV-2 convalescent samples in Cambodia.

## Conclusions

We found in a widely used, highly specific, and validated ELISA that ≈4%–14% of prepandemic serum samples from malaria-infected persons in Cambodia were positive for nonneutralizing antibodies to SARS-CoV-2 spike and RBD antigens by using various standardized optical density cutoff values (*4*,*12*,*13*). We noted a relationship between increased SARS-CoV-2 seroreactivity and antimalarial humoral immunity, which was also recently shown in Africa (S. Lapidus et al., unpub. data). The plausibility of regular spillover events, or simply increased exposure to uncharacterized betacoronaviruses, as a reason for SARS-CoV-2 cross-reactivity is also increased in settings at high risk for zoonotic disease transmission because of agricultural and dietary practices such as bat guano collection and consumption of wild meats (*9*–*11*). Given that 50%–80% of GMS residents are classified as rural, careful calibration of serologic assays targeting SARS-CoV-2 will be necessary in national and subnational serosurveys. Although neutralization assays with live virus are often considered the standard because of their specificity, they are cost-prohibitive for large-scale serosurveys. The use of competition ELISA assays such as surrogate virus neutralization tests targeting the RBD-ACE2 blockade might be an attractive option for populations at high risk for zoonotic exposures in resource-scarce settings without Biosafety Level 3 facilities.

Appendix Additional information about SARS-CoV-2 cross-reactivity in prepandemic serum from rural malaria-infected persons, Cambodia
