# Supplementary material for: SARS-CoV-2 Cross-Reactivity in Prepandemic Serum from Rural Malaria-Infected Persons, Cambodia
Source: Emerg Infect Dis. 2022 Feb;28(2):440–4. doi: 10.3201/eid2802.211725 (PMC8798695; doi:10.3201/eid2802.211725)
Supplement: Appendix — Additional information about SARS-CoV-2 cross-reactivity in prepandemic serum from rural malaria-infected persons, Cambodia [file 21-1725-Techapp-s1.pdf]

Article DOI: https://doi.org/10.3201/eid2802.211725

SARS-CoV-2 Cross-Reactivity in Prepandemic Serum from Rural Malaria-Infected Persons, Cambodia

Appendix

Appendix Table 1. SARS-CoV-2 seroreactivity in rural malaria-experienced persons by site, year, sex, and age, Cambodia*

Site	Samples	Year	Male	Female	<10 y	10–17 y	>18 y
Preah Vihear	81 (15)	2011	51 (9.7)	30 (5.7)	7 (1.6)	27 (6.0)	47 (10.5)
Pursat	347 (66)	2005<br>2009<br>2010<br>2011	261 (49.4)	86 (16.3)	20 (4.5)	71 (15.9)	256 (57.3)
Ratanakiri†	100 (19)	2011	11 (2.1)	8 (1.5)	6 (1.3)	7 (1.6)	6 (1.3)
Total	528	528	323 (61.2)	124 (23.5)	33 (7.4)	105 (23.5)	309 (69.1)

*All values are no. (%) unless otherwise indicated. SARS-CoV-2, severe acute respiratory syndrome coronavirus 2.
†81 samples were missing data on sex and age.

Appendix Table 2. Seropositivity status by 2 SARS-CoV-2 IgG assays in rural malaria-experienced persons, Cambodia*

Participant no.	Test 1 Spike / RBD 2 SD cutoff	Mean RBD OD (450nm)	Mean Spike OD (450nm)	Test 2 Genscript / Spike S1-RBD Cutoff†	Mean (450nm)	Mean (450nm)/ Cutoff (0.12) >1
1	Negative	0.2157	0.2637	Negative	0.0000	0.0000
2	Negative	0.0813	0.1241	Negative	0.0005	0.0041
3	Negative	0.0847	0.3078	Negative	0.0220	0.1812
4	Negative	0.0480	0.0532	Negative	0.0077	0.0630
5	Negative	0.0854	0.5203	Negative	0.0081	0.0663
6	Negative	0.0616	0.1983	Negative	0.0221	0.1820
7	Negative	0.0613	0.1358	Positive	0.2514	2.0708
8	Negative	0.0369	0.0361	Negative	0.0215	0.1767
9	Positive	3.7444	0.7881	Positive	1.0889	8.9691
10	Negative	0.1912	0.2379	Negative	0.0112	0.0923
11	Negative	3.8750	0.4312	Positive	0.6211	5.1161
12	Negative	3.8917	0.4280	Positive	0.6598	5.4349
13	Negative	3.8971	0.3964	Positive	0.7262	5.9815
14	Negative	3.0869	0.3910	Positive	0.4061	3.3451
15	Positive	3.8931	0.9565	Negative	0.0825	0.6796
16	Positive	3.8532	0.9306	Negative	0.0925	0.7615
17	Positive	2.5646	0.7263	Positive	0.2392	1.9703
18	Positive	3.8824	1.9920	Positive	0.4771	3.9296
19	Positive	3.4743	0.9039	Positive	0.5926	4.8814
20	Negative	2.2409	0.6454	Positive	0.2270	1.8694
21	Positive	3.8911	1.9976	Positive	0.6885	5.6713
22	Negative	0.2088	0.2645	Negative	0.0517	0.4255
23	Negative	0.2221	0.1648	Negative	0.0071	0.0585
24	Negative	0.1635	0.1754	Negative	0.0089	0.0729
25	Negative	0.2032	0.2628	Negative	0.0428	0.3526
26	Positive	3.9031	1.5984	Positive	1.3099	10.7900
27	Negative	0.2109	1.0731	Negative	0.1066	0.8777
28	Negative	0.2123	0.2391	Negative	0.0813	0.6697
29	Negative	0.1325	0.1466	Negative	0.0050	0.0412
30	Positive	3.7932	0.9603	Positive	0.6937	5.7142
31	Positive	2.3932	1.5027	Positive	0.2116	1.7430
32	Negative	0.2703	0.3878	Negative	0.1121	0.9234
33	Negative	0.2216	0.3375	Negative	0.0015	0.0124

Participant no.	Test 1 Spike / RBD 2 SD cutoff	Mean RBD OD (450nm)	Mean Spike OD (450nm)	Test 2 Genscript / Spike S1-RBD Cutoff†	Mean (450nm)	Mean (450nm)/ Cutoff (0.12) >1
34	Negative	3.6172	0.4099	Negative	0.0972	0.8007
35	Positive	3.1024	0.7545	Positive	0.6504	5.3575
Cutoff					0.1215	
Negative control				Negative	0.0014	
Positive control				Positive	2.0219	

*RBD, receptor binding domain; SARS-CoV-2, severe acute respiratory syndrome coronavirus 2.
†Positives for test 2 genscript = mean OD 450/cutoff (0.12) >1.

Appendix Table 3. SARS-CoV-2 surrogate virus neutralization test results in rural malaria-experienced persons, Cambodia*

Participant no.	Mean OD sVNT assay	Mean/ Positive CTRL	(1-Mean/Positive CTRL)	% neutralizing capacity	Detectable SARS-CoV-2 neutralizing antibody	Mean RBD	Mean Spike	Spike/RBD seropositive
1	2.9965	1.3873	−0.3873	−38.73	NO	0.2157	0.2637	Negative
2	2.9837	1.3814	−0.3814	−38.14	NO	0.0813	0.1241	Negative
3	3.0597	1.4166	−0.4166	−41.66	NO	0.0847	0.3078	Negative
4	3.0404	1.4076	−0.4076	−40.76	NO	0.048	0.0532	Negative
5	2.8734	1.3303	−0.3303	−33.03	NO	0.08545	0.5203	Negative
6	2.9994	1.3886	−0.3886	−38.86	NO	0.06165	0.1983	Negative
7	3.0158	1.3962	−0.3962	−39.62	NO	0.0613	0.1358	Negative
8	2.8516	1.3202	−0.3202	−32.02	NO	0.03685	0.0361	Negative
9	2.2910	1.0607	−0.0607	−6.07	NO	3.74435	0.7881	POSITIVE
10	2.8065	1.2993	−0.2993	−29.93	NO	0.1912	0.2379	Negative
11	2.2671	1.0496	−0.0496	−4.96	NO	3.87495	0.4312	Negative
12	2.3722	1.0982	−0.0982	−9.82	NO	3.8917	0.428	Negative
13	2.5024	1.1585	−0.1585	−15.85	NO	3.8971	0.3964	Negative
14	2.5129	1.1634	−0.1634	−16.34	NO	3.08685	0.391	Negative
15	2.4956	1.1554	−0.1554	−15.54	NO	3.89305	0.9565	POSITIVE
16	2.5930	1.2005	−0.2005	−20.05	NO	3.85315	0.9306	POSITIVE
17	2.2649	1.0486	−0.0486	−4.86	NO	2.56455	0.7263	POSITIVE
18	2.5655	1.1878	−0.1878	−18.78	NO	3.8824	1.992	POSITIVE
19	2.2631	1.0477	−0.0477	−4.77	NO	3.47425	0.9039	POSITIVE
20	2.4560	1.1371	−0.1371	−13.71	NO	2.2409	0.6454	Negative
21	1.8539	0.8583	0.1417	14.17	NO	3.8911	1.9976	POSITIVE
22	2.8783	1.3326	−0.3326	−33.26	NO	0.2088	0.2645	Negative
23	2.8563	1.3224	−0.3224	−32.24	NO	0.2221	0.1648	Negative
24	2.6625	1.2326	−0.2326	−23.26	NO	0.1635	0.1754	Negative
25	2.7740	1.2843	−0.2843	−28.43	NO	0.20315	0.2628	Negative
26	2.4614	1.1396	−0.1396	−13.96	NO	3.90305	1.5984	POSITIVE
27	2.8388	1.3143	−0.3143	−31.43	NO	0.21085	1.0731	Negative
28	2.7149	1.2569	−0.2569	−25.69	NO	0.2123	0.2391	Negative
29	2.6724	1.2373	−0.2373	−23.73	NO	0.13245	0.1466	Negative
30	2.2980	1.0639	−0.0639	−6.39	NO	3.79315	0.9603	POSITIVE
31	1.9825	0.9178	0.0822	8.22	NO	2.39315	1.5027	POSITIVE
32	2.1600	1.0000	0.0000	0.00	NO	0.27025	0.3878	Negative
33	2.8539	1.3213	−0.3213	−32.13	NO	0.22155	0.3375	Negative
34	2.4904	1.1530	−0.1530	−15.30	NO	3.6172	0.4099	Negative
35	2.3534	1.0895	−0.0895	−8.95	NO	3.10235	0.7545	POSITIVE
Neg. CTRL	2.5788	1.1939	−0.1939		NO			
Pos. CTRL	0.0863	0.0399	0.9601	96.01	YES			
SARS-CoV-2 neutralizing antibody standard serial dilution								
156.25	1.1358	0.5258	0.4742	47.42	YES			
312.5	0.5059	0.2342	0.7658	76.58	YES			
625	0.1636	0.0757	0.9243	92.43	YES			
1250	0.0687	0.0318	0.9682	96.82	YES			
2500	0.0490	0.0227	0.9773	97.73	YES			
5000	0.0465	0.0215	0.9785	97.85	YES			
10000	0.0517	0.0239	0.9761	97.61	YES			

*SARS-CoV-2, severe acute respiratory syndrome coronavirus 2; sVNT, surrogate virus neutralization test.

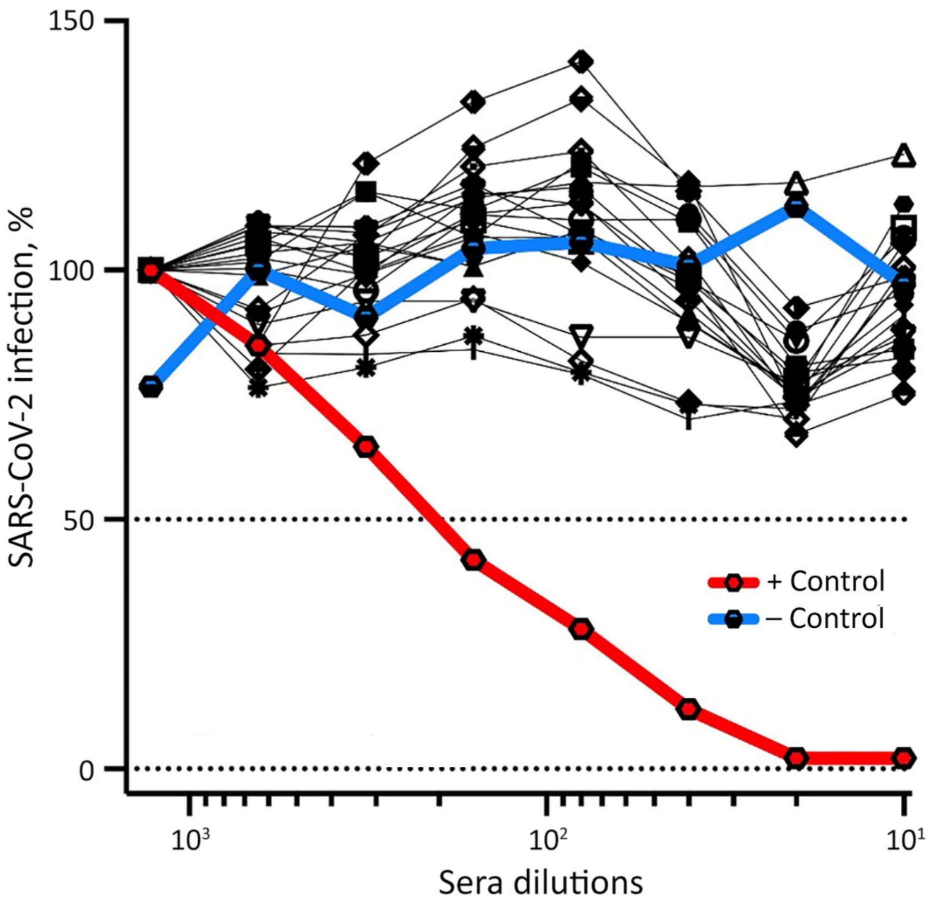

Appendix Figure. Microneutralization of SARS-CoV-2 in serum samples from pre-pandemic malaria-experienced rural persons, Cambodia. Twenty-one SARS-CoV-2 ELISA-positive Cambodian serum samples, negative controls (serum samples from US patients in 2014), and positive controls (serum samples from US patients who died of SARS-CoV-2 infection in 2020) were heat-inactivated (56°C, 1hr), serially 2-fold diluted (1:10 to 1:1,280) in OptiPRO SFM (catalog no. 12309–019, ThermoFisher Scientific, https://www.thermofisher.com) supplemented with 2mM L-Glutamine (catalog no. 25030–081, ThermoFisher Scientific) and 1x Antibiotic-Antimycotic (catalog no. 15240062, ThermoFisher Scientific). Diluted serum samples were mixed with an equal volume of SARS-CoV-2 diluted to 200 TCID50/25µl (USA-WA1/2020, catalog no. NR-52281, BEI Resources, https://www.beiresources.org) and incubated at room temperature for 1 hour. Fifty micro liter of the virus-plasma mixture was added in triplicate to Vero cells grown in a 96-well plate and incubated for 3 days at 37°C in a humidified incubator with 5% CO2. After incubation, media was removed and 200µL of 10% neutral buffered formalin was added and incubated at room temperature for 30 min to inactivate the virus and fix the cells. After incubation, the plates were washed 3 times with wash buffer (0.05% Tween 20 in PBS), and 1:4,000 diluted SARS-CoV-2 nucleocapsid antibody (catalog no. 40143-R001, Sino Biological, https://www.sinobiological.com) was added to each well (50 µL per well). After incubation (room temperature, 1hr), the plates were washed 3 times, and 1:10,000 diluted horseradish peroxidase-conjugated anti-rabbit IgG antibody (catalog no.

32460, ThermoFisher Scientific) was added (100 µl per well), and the plates were incubated at room temperature for 1 hr. The plates were then washed 6 times followed by 30 min of room temperature incubation with horseradish peroxidase substrate solution (100 µL per well) prepared by adding a 10-mg o-phenylenediamine dihydrochloride tablet (catalog no. P8287, MilliporeSigma, https://www.sigmaaldrich.com) to 20 mL of phosphate citrate buffer preparation (catalog no. P4922, MilliporeSigma). The reaction was stopped by adding 1 M sulfuric acid (100 µL per well), and the optical density was measured at 492 nm (OD492). To calculate percent infection, the optical density obtained with the lowest amount of serum (1:1,280) was used to set 100% infection for each serum sample. SARS-CoV-2, severe acute respiratory syndrome coronavirus 2.
